# Implementation of Teleophthalmology to Improve Diabetic Retinopathy Surveillance: Qualitative Interview Study of Clinical Staff Informed by Implementation Science Frameworks

**DOI:** 10.2196/32162

**Published:** 2022-03-30

**Authors:** Rajeev S Ramchandran, Reza Yousefi-Nooraie, Porooshat Dadgostar, Sule Yilmaz, Jesica Basant, Ann M Dozier

**Affiliations:** 1 Flaum Eye Institute University of Rochester Medical Center Rochester, NY United States; 2 Department of Public Health Sciences School of Medicine and Dentistry University of Rochester Rochester, NY United States; 3 Division of Supportive Care in Cancer Department of Surgery University of Rochester Medical Center Rochester, NY United States

**Keywords:** Consolidated Framework for Implementation Research, teleophthalmology, diabetic retinopathy, implementation, qualitative study, Practical, Robust Implementation and Sustainability Model

## Abstract

**Background:**

The store-and-forward camera-based evaluation of the eye, or teleophthalmology, is an effective way to identify diabetic retinopathy, the leading cause of blindness in the United States, but uptake has been slow. Understanding the barriers to and facilitators of implementing teleophthalmology programs from those actively adopting, running, and sustaining such programs is important for widespread adoption.

**Objective:**

This study aims to understand the factors that are important in introducing teleophthalmology to improve access to diagnostic eye care for patients with diabetes in primary care clinics by using implementation science.

**Methods:**

This qualitative study in 3 urban, low-income, largely racial and ethnic minority–serving safety-net primary care clinics in Rochester, New York, interviewed nurses and physicians on implementing a teleophthalmology program by using questions informed by the Practical, Robust Implementation and Sustainability Model and the Consolidated Framework for Implementation Research.

**Results:**

Primary care nurses operationalizing the program in their clinics saw increased work burden and a lack of self-efficacy as barriers. Continuous training on the teleophthalmology process for nurses, physicians, and administrative staff through in-service and peer training by champions and superusers were identified by interviewees as needs. Facilitators included the perceived convenience for the patient and a perceived educational advantage to the program, as it gave an opportunity for providers to discuss the importance of eye care with patients. Concerns in making and tracking referrals to ophthalmology because of challenges related to care coordination were highlighted. The financial aspects of the program (eg, patient coverage and care provider reimbursement) were unclear to many staff members, influencing adoption and sustainability.

**Conclusions:**

Streamlining processes and workflows, training and assigning adequate staff, effectively coordinating care between primary care and eye care to improve follow-ups, and ensuring financial viability can all help streamline the adoption of teleophthalmology.

## Introduction

### Background

Diabetic retinopathy is the leading cause of blindness in working-age US adults, resulting in high personal, social, and economic costs [1,2]. Although having an annual dilated eye examination can timely identify vision-threatening disease and avoid blindness with timely treatment in ≥95% of patients with diabetes, annual dilated retinal examination rates are still <50%, especially for those with low income and who are uninsured or underinsured [3,4]. Teleophthalmology for diabetic retinopathy surveillance (DRS) is the store-and-forward process of remotely evaluating patients with diabetes for retinopathy. It involves placing digital nonmydriatic retinal cameras in nonophthalmic health care settings and linking them to eye care providers via telecommunication technology such as the internet [5-16]. Notably, ubiquitous screening programs in the United Kingdom have helped replace diabetic retinopathy with inherited eye diseases as their leading cause of blindness (>90% of patients with diabetes have an annual eye examination or retinal screen) [17].

Although the effectiveness of teleophthalmology to substantially increase annual retinal screening rates for vision-threatening diabetic retinopathy at primary care clinics caring for low-income populations is well-established, sustained implementation is challenging [18]. Those programs funded through grants or philanthropic support may not be sustainable once initial funding ends. Moreover, not all clinics are willing to initiate or adopt telemedicine-based DRS. Currently, the implementation of teleophthalmology for DRS uses a trial-and-error process. Generalized knowledge is needed to implement and sustain teleophthalmology programs to avoid each new group “re-inventing the wheel” [18].

### Teleophthalmology Implementation

Implementation science is the systematic study of strategies to adopt and integrate evidence-based [19] approaches into real-world practice. Frameworks and models from implementation science can be tailored to effectively study how and why teleophthalmology programs are accepted and sustained in some clinics but not in others [20]. To date, the published literature has focused on reporting standard outcomes of teleophthalmology for DRS programs that have increased annual rates of examining eyes for vision-threatening retinopathy [21] rather than on implementation. Specific outcome measures have included changes in the number of patients screened, the number who followed up to eye care, demographics, modeled costs, and patient satisfaction [5,6,8-18,21-25]. A recent study focused on implementing teleophthalmology explored how rural primary clinics in Wisconsin viewed the implementation of teleophthalmology using qualitative analysis and an implementation science framework [26]. Another study focused on the implementation of teleophthalmology across federally qualified health centers in Kentucky using implementation science metrics [27]. In this study, we report on the implementation of a teleophthalmology program for diabetic retinopathy and visual acuity surveillance in urban, low-income, largely racial and ethnic minority–serving primary care clinics in Rochester, New York, using implementation science frameworks.

## Methods

### Ethics Approval

The Research Subjects Review Board of the University of Rochester approved this study (approval number RSRB00065090). Given the activities and nature of the study, the Research Subjects Review Board deemed that verbal consent was sufficient for participating in the study, and all interviewees provided informed verbal consent and agreed to have their interview audio-recorded. A description of the teleophthalmology program has been previously published [28].

### Participants, Setting, and Description of the Intervention

Providers and staff from 3 safety-net primary care clinics that cared for low-income, uninsured, and underinsured largely racial and ethnic minority populations (both Hispanic and African American) and had implemented teleophthalmology to increase retinal evaluations for their patients with diabetes were invited to participate in a semistructured interview regarding their experience with program implementation. The research team then followed up with each interested participant to schedule a face-to-face interview based on participant availability. All primary care clinics were teaching sites for trainees in medicine and were staffed by attending physicians.

The 3 clinics cared for between 550 and 1250 patients with diabetes and had annual eye examination rates for this population of 20% to 40%, which doubled after the implementation of teleophthalmology. A Zeiss Visucam NM PRO (Zeiss) nonmydriatic fundus camera was used in 67% (2/3) of the clinics, and the Topcon NW400 (Topcon) nonmydriatic fundus camera was used in the third clinic. The teleophthalmology program used software that was developed internally by the ophthalmology department to capture data on the patients who were evaluated via teleophthalmology. This system required the manual entry of patient information, such as name, date of birth, and demographics. This system remained outside the electronic medical record system routinely used by the clinics. Staff were trained on the cameras and teleophthalmology program software by ophthalmology staff and signed off on being proficient at using the cameras and assessing vision for the program. The training was held at the beginning of the program and was repeated every 3-4 months as necessary when new staff joined the clinics.

During the imaging training and in subsequent refresher sessions, staff were trained on recognizing the difference between readable and unreadable images. An image quality comparison guide was provided during training to indicate which factors had the potential to compromise image clarity resulting in an unreadable image. The most commonly seen factors were shadows; dust, dirt, or other camera lens opacities; haze; artifacts; and small pupils. Unreadable images as graded by a retina specialist (RSR) accounted for 11% (69/627) of the patients assessed with the camera at the time the staff were interviewed. Photographers were given feedback on image quality as specified on each report to ensure staff captured readable images. Image quality was graded by RSR as *poor*, *adequate*, *good*, *fair*, and *excellent*, and the factors that influenced the image grade were included in the report provided to the clinics. Providing image grading allowed photographers the opportunity to re-evaluate their techniques and make continuous improvement on capturing readable images.

The results and recommended follow-up directions to see an ophthalmologist by RSR, who reviewed the images and patient data, were sent to the primary care office, who then contacted the patients. Notification that a report was ready to be downloaded was sent by email to the primary care clinic contact, and the report was accessed via a web-based portal by the clinic staff. The clinic staff added these reports to the patient’s electronic medical record. The primary care office also communicated the results of screenings to the patients and notified patients regarding when they needed to follow up for further eye care. These results were shared with the patient via phone by the primary care clinic within a week of the camera-based eye evaluation. The program in 67% (2/3) of the clinics was grant-funded, and patients were not billed for the digital camera images taken of their eyes. Patients receiving teleophthalmology in the third clinic may have been billed for having images of their eyes taken, but billing was inconsistent as it was not routinely monitored [22].

### Data Collection

The participants who were involved in championing, implementing, and day-to-day operations of the teleophthalmology program at each clinic were emailed the interview questions in advance of the face-to-face interviews. They were also given the option to complete the questions via email. Of the 11 participants, 1 (9%; a primary clinic physician) chose this option, and the remaining 10 (91%) were interviewed. The interviews were conducted by research staff from August to October 2017 in 3 primary care settings. Each interview lasted approximately 15 to 20 minutes and was audiotaped. The participants provided verbal consent for audio-recording and could decline to answer any question. The recordings were then transcribed word-for-word by a professional transcriptionist.

The interview questions were selected from the adapted Practical, Robust Implementation and Sustainability Model (PRISM) [29] and Consolidated Framework for Implementation Research (CFIR) [30]. These questions included items related to the intervention characteristics (eg, adaptability, trialability, and complexity), characteristics of the individuals (eg, knowledge and beliefs about the intervention), the organization or inner setting (eg, infrastructure, resources, workflows, and supports), the external environment or outer setting (eg, patient beliefs, financial barriers, and reimbursement mechanism), the process of implementation (eg, engaging, executing, and reflecting and evaluating), and sustainability (eg, structural characteristics, implementation climate, readiness for implementation, resources, and modifications needed for sustainability; see Multimedia Appendix 1 for the interview guide). The interview guide served to direct the conversation, but the interviewees were able to discuss other issues that were not included in the guide.

### Data Analysis

Thematic codes about program implementation were generated from the interview data using the PRISM and CFIR frameworks. Authors (RR, RYN, and SY) coded the transcripts based on an initial coding framework informed by the CFIR and PRISM. The coding framework expanded inductively through the coding process to address new themes not originally included. All authors discussed all coded segments in regular meetings and reached a consensus on the structure of emerging themes. This step entailed a rigorous back-and-forth comparison of data against the elements of the PRISM and CFIR frameworks and other emerging themes. Although both frameworks were considered to inform the analysis, we used the CFIR to organize the results and frame various determinants of the implementation. The PRISM mostly informed themes related to the organizational resources and infrastructures as well as considerations on sustainability. Although implementation frameworks informed the thematic analysis, we paid special attention to themes pertaining to other considerations not covered by these 2 frameworks. For example, we incorporated themes related to training staff, leadership support, and the role of champions as important ingredients of implementation success.

## Results

### Overview

Over 1 year of implementing the teleophthalmology program, each clinic doubled its annual retinal examination rate for patients with diabetes.

The project began as a community service quality improvement pilot project at 67% (2/3) of the clinics before the fee-for-service billing for the intervention was considered. Fee-for-service billing was implemented at the third primary care clinic upon starting the teleophthalmology program as this clinic was in the same health system as the partner ophthalmology department, and billing for the technical and professional component using the 99250 Current Procedural Terminology code was conducted for the ophthalmic photographs taken as part of the teleophthalmology program [22]. Of the 14 clinic staff members who were contacted, 11 (79%) agreed to participate in the semistructured interviews across all 3 sites. This group included 3 primary care physicians (medical doctors; 3/11, 27%), 1 pharmacist (1/11, 9%), 1 nurse practitioner (NP; 1/11, 9%), 1 administrator (1/11, 9%), and 5 registered nurses (RNs; 5/11, 45%) who ran the day-to-day operations and were responsible for the daily workflow of the teleophthalmology process. Of the other 3 clinic staff members invited to participate, 1 (33%) RN reported not having direct experience with the teleophthalmology program, and the other 2 (67%), both RNs, did not respond.

Through the qualitative analysis of the staff interviews, we identified five main categories of themes related to the implementation of teleophthalmology: individuals involved in the implementation, characteristics of the intervention (technology and tasks), process of implementation, and characteristics of the inner and outer setting or environment of the primary care clinic. Not surprisingly, respondents involved in day-to-day operations (RNs) noted specific operational and logistical aspects of program implementation, whereas those who were less involved (providers or administrators) noted more general and system-level aspects of program implementation. Table 1 provides the classification of themes with relevant quotes. These main themes are discussed in detail in the following sections.

**Table 1:** Qualitative themes and supporting quotes^a^.

Category and themes				Supporting quotes
**Individuals and inner setting**				
	**Patients**			
		Convenience	“Patients are glad the photo can be done at the same place...the photo is quick. They often say thank you and that [they’ve] been meaning to [get screened].” [Participant 10, NP^b^] “Issue comes when the patient has other things that get in the way [or] they’re not able to follow instructions well.” [Participant 1, RN^c^] “We have taken pictures of patients who’ve never been to an eye doctor before.” [Participant 9, RN]	
		Patient communication and arrangement	“It’s hard to get our patients in for a visit period, but when they’re in for a visit and it’s already taking long and then you have to do the eye screen afterwards, they may not have allotted themselves that much time here at clinic.” [Participant 5, RN] “Letting the patient know ahead of time [that they] have an opportunity to get an eye screen [important].” [Participant 2, PharmD^d^]	
	**Staff and inner setting**			
		Motivation and buy-in	“[Using the camera] made my job more enjoyable.” [Participant 3, RN] “It’s cool to see the eye.” [Participant 9, RN]	
		Limited resources (time and staff)	“Even though the procedure itself doesn’t take that long, to try to fit it in with a staff that’s competent to do the screening [is a problem].” [Participant 6, NP, and participant 12, administrator] “...Some slow buy-in by the nurses because then they were feeling like we’re short staffed.” [Participant 6, NP] “...Currently short staffed three nurses [making it hard to support program].” [Participant 10, NP] “...Challenge implementing, taking time and staff away from the normal flow to get it done.” [Participant 8, MD^e^]	
**Characteristics of the intervention**				
	Camera ease of use		“It’s intimidating by looking at the machine, but it’s actually a lot easier than it looks.” [Participant 1, RN]	
	Technology and workflow complexity		“[Technology didn’t] work all the time, when operational it’s great...You can send the results right away to [ophthalmology].” [Participant 9, RN] “As routine...internal processes have been developed...entire screening process...reduced to [about] 10 minutes.” [Participant 10, NP]	
	Referral and follow-up with eye care		“How you make a referral is the more challenging part...don’t have resources to be tracking every referral.” [Participant 3, MD] “...Nice if there was [more] follow-up from [ophthalmology department] to close the loop [with us].” [Participant 10, NP] “...Biggest challenge to long-term sustainability is maintaining that relationship between 2 different departments.” [Participant 3, MD] “[Other similar programs exist in] New York State...but not as coordinated as we are doing it with Ophthalmology.” [Participant 3, MD] “[Ophthalmology Program staff]...good about follow-up and checking in.” [Participant 11, MD]	
**Implementation processes**				
	Education and training		“[What] motivates nurses is [regular] in-services [teaching] the importance of eye health [and use of system].” [Participant 1, RN] “[Initial eye health, diabetes, and camera demo talks] engaged the physicians and the residents in the process.” [Participant 3, MD] “...cause we have so many resident physicians that if we had an in-service showing how important that eye health is then and how we have this machine and its capabilities I feel like it would get used so much more.” [Participant 3, MD] “...Brings PCPs^f^ right into the mix, so there’s a lot of benefits for the providers. Since we are a resident training clinic, I think there is a huge educational benefit.” [Participant 3, MD] “[We] felt competent to develop the workflow, and [use] the machine. We tried to solve barriers, but without nurse to staff it [we] added training for residents.” [Participant 11, MD]	
	Hands-on experience		“Yeah...everybody really enjoyed having it here. We tested a lot on the employees to just get the hang of things.” [Participant 4, RN]	
	Champions		“...Champions at site is key.” [Participant 3, MD] “[Champions who train others]...who know the program leaving clinic.” [Participant 9, RN] “It seemed like the reimbursement was very low so that part was difficult to make sustainable.” [Participant 8, MD]	
**Outer setting**				
	Awareness and attitude		“Some people just do not care [about their eyes]. Hopefully this program provides a prompt to keep up with eye care if they are not already doing so.” [Participant 10, NP] “Patients [don’t] understand the gravity of how diabetes can affect their eye health...more education...need[ed].” [Participant 5, RN]	
	Financial		“Well, a lot of our patients are Medicaid but I have no idea how it works on the insurance side of it.” [Participant 1, RN] “[For] patients that don’t have any insurance, we have a program ‘charity care’...cover [the costs].” [Participant 3, MD]	

^a^Who said each statement is identified at the end of each quote.

^b^NP: nurse practitioner.

^c^RN: registered nurse.

^d^PharmD: Doctor of Pharmacy.

^e^MD: Doctor of Medicine.

^f^PCP: primary care provider.

### Individuals and Inner Setting

This theme focuses on the characteristics, skills and self-efficacy, motivation, and perception of resources, namely time, by staff and patients.

#### Staff and Inner Setting of the Primary Care Clinics

Inner setting per the CFIR framework includes structural and cultural context through which the implementation process takes place [30]. In our analysis, we merged the themes related to staff characteristics with the inner setting in which they were embedded because of the substantial overlap between concepts.

Staff noted that having the program at their clinic brought a meaningful, novel way to help their patients. They were excited to have the camera and believed in the program’s utility to screen their patients for retinopathy and vision loss in their clinics. This belief was a strong motivator and provided buy-in at the individual level to adopt this program for patients with diabetes. RN staff most often had comments about the daily workflow and camera use. As expected with the adoption of new technology or innovation, there was some preliminary hesitation because of perception of the technology, additional workflow elements, and preconceived notions of the complexity of the technology. However, once staff had training and a chance to use the camera on their own, they found that they could be more efficient at screening with the camera. Staff also saw value in being able to provide improved access to eye care as they could expeditiously obtain a review of retinal images and vision-screening information by an ophthalmologist for their patients and thereby reduce by at least 6 months the overall wait time for patients to see an ophthalmologist to obtain timely sight-saving treatment.

Another facilitator of program acceptance was a perceived educational advantage to the program in that it allowed primary care providers to discuss eye care as an ongoing topic with patients. Having the camera in the clinic increased conversations around eye care in patients with diabetes, especially among staff and resident physician trainees, and was something the practices would show off to their resident interviewees as an innovative intervention to help improve access to care.

Staff did express facing challenges with supporting the program as nurses felt that they were short-staffed and they did not always have the time to incorporate the teleophthalmology workflow into their typical workflow. An NP and physicians also frequently cited time constraints and nursing staff allocation as challenges to implement the program. Nursing staff specifically expressed that they did not have the time and resources to support the program as communicated. These comments highlight the anxiety generated among staff that felt that they had limited capacity and bandwidth to take on new responsibilities.

#### Patients

We have previously reported on patient perspectives obtained from surveys and focus groups [28]. Interviewed staff felt that their patients saw the camera-based evaluation as a convenient and valuable benefit for their health. Staff also commented that patients were grateful to have such a program in their primary care clinic as they had been putting off obtaining an eye examination with an ophthalmologist because of other priorities. Staff further noted that patients were more motivated to participate when they felt the images were captured quickly and conveniently and when their primary care providers recommended the teleophthalmology evaluation. The screening did not seem to take long per the staff but did add additional time to visits. In addition, finding an available staff member who was trained to carry out the teleophthalmology evaluation was difficult at times as these staff members were occupied by other clinical tasks. Previous communication with patients about the longer process was also noted as important to staff. Patients may not have been prepared for the longer visit duration and often did not anticipate staying the extra 10 to 15 minutes needed to complete the teleophthalmology evaluation, which was mostly done at the end of the primary care visit.

### Characteristics of the Intervention

This category included themes related to technology, tasks, and workflows. Staff believed that the usability of the camera was not a significant challenge. Nursing staff noted that the nonmydriatic camera seemed intimidating at first but was easy to use and worked well, although the camera software did not function smoothly all the time. A key facilitator of program adoption was that staff felt that the program was helpful in that evaluation of the eye for diabetes-related eye disease was available during the patient’s primary care provider visit. Once an internal process to improve the workflow of the teleophthalmology project had been established, the screening process was reduced to approximately 10 minutes.

Tasks and workflows were seen as a significant challenge from the staff’s perspective. The tasks included scheduling and patient notification, data entry into the teleophthalmology software, and tracking of the ophthalmologist referral. Both physicians and nurses said that nurses were an integral part of the workflow and that all nurses who could potentially conduct the camera-based evaluation should learn the workflow and process. In reality, there were certain superusers and a limited number of nursing staff assigned by the clinics for training in the teleophthalmology process, including using the camera.

Follow-up to eye care also remained a challenge. Staff expressed concerns in making and tracking each referral to ophthalmology as they did not have the necessary resources to do so. All cadres of staff felt that maintaining a long-term relationship between primary care and ophthalmology could pose a challenge in the future as they felt that there was not enough follow-up from the ophthalmology department to close the loop with the clinic; however, they acknowledged that the proponents of the program in ophthalmology worked at maintaining good communications and relationships.

### Implementation Processes

**Figure 1:**
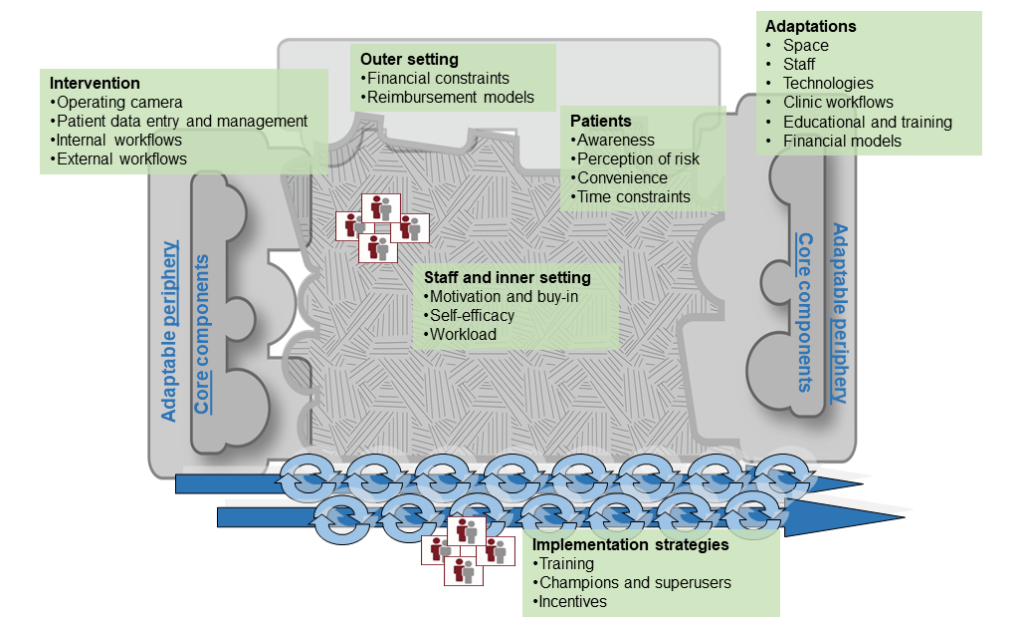
Mapping qualitative themes onto Consolidated Framework for Implementation Research domains (adapted from Damschroder et al [30]).

This category included activities carried out at different levels to help establish the technology and teleophthalmology process in the clinic and incorporate it as part of routine care (Figure 1). It involved training and assigning champions, staff training to perform the teleophthalmology process, increasing awareness and visibility of the program among clinic staff and patients, and the process of referring patients who used teleophthalmology to ophthalmology from primary care at the recommended interval per their evaluation.

#### Training

Staff found that providing in-service training to learn the importance of eye health was key to keeping physicians and residents engaged in the process. In some clinics, staff complemented formal training with hands-on experience with peers and found this additional practice with the technology and process helpful. The training of multiple staff in conducting the teleophthalmology process, including becoming very familiar with operating the camera and the web-based data entry platform, especially as new staff were hired by the clinic, was repeatedly mentioned as an essential aspect of implementing the intervention. Both the intensity and scope of training were cited as important. Physicians and nurses also viewed training and raising awareness of the program for primary care clinical, administrative, clerical, and physician staff as important.

Scheduling and front-desk clinic staff were responsible for contacting patients for a primary care appointment during which the camera-based evaluation would also be done. Thus, making this staff aware of the rationale and process of what the patient would experience so that they could clearly communicate this to the patient during conversations occurring for scheduling and checking in the patient to the clinic was deemed important by both nurses and physicians. Nurses and physicians also agreed that physicians, including residents, and NP providers should be made aware of the availability of the camera-based evaluation and of the process so that they could counsel their patients who needed such an examination on the process and need to have the evaluation before they left the clinic. Respondents felt that training should include both hands-on use of the camera and running through the workflow of the teleophthalmology evaluation process, along with didactic education on the effects of diabetes on the eye. Overall, personalized, live-human training was preferred. Respondents in all clinics requested periodic in-services and refresher sessions on using the camera, on how the program fit in the daily workflow, and on the rationale of performing the examinations in all clinics.

#### Champions

Another crucial implementation strategy was the identification and recognition of champions, who were superusers [31]. At each site, at least one RN was identified and trained as a superuser. These superusers were very adept at using the camera and electronic workflow, as deemed by the ophthalmology implementation team, before implementing the program. The superuser would train others in the clinic and champion the program by ensuring that patients were identified and taken through the camera-based eye examination process. When these champions were involved in the program, uptake was strong. However, these champions often did not remain at the clinic for >6 months. There was also a high turnover of clinic nurses, which also included those trained to use the camera and the electronic workflow by the ophthalmology implementation team and superuser RN. This high turnover was cited as a challenge to implementation by all cadres of staff.

#### Leadership

Leadership buy-in and motivation to implement the teleophthalmology program within the clinical setting served as vital components to achieve the support for workflow changes and sustainability of the program. Primary care physician leaders in the clinics were key to implementation. They helped emphasize the need for performing eye examinations to their colleagues, who were more apt to discuss the need for the examination with their patients. Physicians helped facilitate patient acceptance of teleophthalmology by discussing the importance of having an eye examination to determine their level of eye disease and describing the convenience of having the camera-based evaluation of their eye in lieu of an immediate eye examination with an ophthalmologist while they had come for their primary care visit. The physicians themselves saw the need for leadership buy-in across departments and medical specialties. Understanding how ophthalmology and primary care would work together to address patient needs and support the teleophthalmology initiative was one of the biggest challenges. Having physician champions translated into having administrative buy-in as physicians were often administrators themselves or had good relationships with nonphysician administrators. In addition, nonphysician administrators, who saw their role of helping physicians deliver quality care, also valued the teleophthalmology program as they felt it was an essential quality improvement program for the clinic.

#### Perceived Benefits

Primary care clinic leadership and administrators noted meeting the Healthcare Effectiveness Data and Information Set metric and elevating the profile of the clinic as a provider of the highest standard of care as 2 reasons for implementing the camera-based eye examinations. Meeting the eye examination metric gave 3 points out of 100 toward meeting the overall score considered in granting various quality distinctions by rating agencies. However, the exact dollar amounts to be gained by meeting these incentives could not be specifically identified. Clinic leadership acknowledged that the program could not be supported by existing monetary incentives and by qualifying as a quality center for health care by offering the program. At the end of the 2-year pilot at the clinics where the intervention was implemented as a community service, there was discussion of sustaining the program with the fee-for-service billing. However, physician champions and administrators at these clinics noted that the reimbursement was too low to make it sustainable, and they decided not to continue with the teleophthalmology program after the grant period ended.

### Outer Setting

This category focused on external factors affecting the process of implementation, including financial constraints and community awareness of eye health. Staff expressed that they did not feel that patients knew the importance of how diabetes affected their eyes, so more education was needed in the community. The lack of awareness and recognition could potentially jeopardize the sustainability of the intervention and patients’ participation in follow-up visits.

Staff noted that financial support was an important consideration, especially in the low-income population that their safety-net clinics served. The nursing and physician staff were not aware of the specific costs or financial implications of the program to the patient as these points were not discussed or fully vetted before the start of the program. Staff noted that financial support was an important consideration for patients, and a physician in the clinic who implemented fee-for-service billing did note that the health system provided funds to cover the health care costs of the teleophthalmology evaluation for those whose income level qualified.

## Discussion

### Principal Findings

#### Overview

This study identified several factors affecting the process of implementing teleophthalmology in primary care using the PRISM and CFIR frameworks. Figure 1 shows the main factors of implementing teleophthalmology identified in this study across CFIR domains [30]. These include the complexity of the teleophthalmology intervention, the need for and feasibility of active implementation strategies (such as training and champions), and the context-specific barriers related to inner and outer settings of primary care. Discussion of these main domains in the context of teleophthalmology occurs in the following sections.

Liu et al [18] conducted a qualitative study involving patients and primary care providers to learn about their experience with the implementation of teleophthalmology in rural primary care clinics. They classified factors associated with teleophthalmology implementation across different workflow stages, including the process of determining patient eligibility, patient referral, and activities during the patient appointment for the teleophthalmology evaluation. The main barriers to implementing teleophthalmology according to Liu et al [18] were the patients’ unfamiliarity and negative attitude toward eye care and logistical challenges in attending their appointments, primary care physician lack of knowledge and data system capabilities in identifying eligible patients and making referrals, and lack of proper communication between patients and care providers. Our findings are consistent with the study by Liu et al [18] in terms of barriers viewed by the provider, such as time constraints and conflicts with existing workflows, which can negatively affect the implementation of the program. This study also complements the framework by Liu et al [18] by focusing on organizational structure and incentives and provides a holistic picture of clinical staff’s experiences and perceptions. However, unlike Liu et al [18], we also interviewed the nurses who actually performed the teleophthalmology workflow in our settings. Thus, our findings may better reflect the perspectives of and challenges experienced by those actually conducting the teleophthalmology program in primary care clinics.

#### The Complexity of the Intervention

This study’s qualitative findings highlighted the complexity of teleophthalmology as an intervention. In recent years, more attention has been paid to disentangling the dimensions of complexity [32] of interventions and developing strategies to facilitate their implementation by addressing the complexity [33]. Complex interventions have several interacting active ingredients and blurred boundaries between the intervention, implementation strategies, and contexts within which the intervention is being implemented [34]. We noted these considerations in our own program evaluation. Implementation of teleophthalmology involves several interacting moving parts, including the camera, its operation and software, the patient health information system, the internal workflow within the clinic, the external workflow between primary care and ophthalmology, and the feedback and follow-up system with patients and eye care. Several contextual factors influence these interacting components, including financial and time-based resource constraints, which affect the success of the implementation. We noted individual characteristics of the staff, human resources and workload, existing physical and information technology infrastructure within the clinic, and existing relationships with the eye care provider clinics as key areas to be addressed in implementing teleophthalmology as identified by primary care clinic providers, administrators, and staff. These are embedded in a larger context of the patient population, their knowledge and readiness, and financial incentives to promote service use, which are limited especially for low-resourced or low-income and, thus, more vulnerable populations. The noticeably increased administrative and staff resources, communication gaps, existing challenges of finding suitable candidates for the program, and heavy dependence on patients’ involvement in the program, especially in terms of follow-up to eye care, have been seen to further complicate the implementation and success of teleophthalmology and are also consistent with the experience of implementing other eHealth interventions [35].

#### The Importance of Champions, Facilitators, and Continuous Training

Champions and superusers were identified in all our clinics to raise awareness of the program and train staff. This was necessary as not all staff could be trained to use the technology during the ophthalmology-led training sessions. Although this strategy usually worked, it induced challenges when the champion was not available, was not recognized by the staff as the go-to person, or left the organization. An alternative solution that was suggested by the interviewees was broader training and continuous engagement of the staff [31]. All staff felt that having educational lectures and hands-on training in a continuous manner, either with regular check-ins and in-person training sessions or by using recorded lectures and training videos on the web, was important. Identification of champions was correlated with improved implementation outcomes [36]. The literature also supports that staff may have more willingness to integrate the program and show interest and commitment to implementation activities after ongoing training [36].

There is growing evidence that champions play a crucial role in the successful implementation and positive outcomes of interventions and are an essential implementation strategy [37]. Thus, the identification, appointment, and preparation of champions contribute to the viability of teleophthalmology programs [31]. Champions require several skills and qualifications and enough motivation to lead the organizational change to be effective in facilitating the implementation [38]. Miech et al [31] recognized >26 different traits for effective champions, varying from being personable and well-liked among peers to having distinguished presentation and communication skills as well as the willingness to engage and lead the efforts according to the program goals and action plan. Champions should be intrinsically motivated and take the initiative in leading the implementation rather than being assigned by the leadership, as was often the case in our clinics, to accomplish their role [37]. Our study found that champions assigned by the clinic administrators of physician leadership sometimes felt overwhelmed or were not intrinsically motivated or recognized by others for their roles. The lack of motivation and organizational recognition might hinder their impact on sustaining the implementation of teleophthalmology in clinics. We suggest that, to facilitate the implementation of teleophthalmology, more attention should be paid to the identification of internal champion staff that self-select into this role and to recognizing the importance of their roles in leading the implementation. As staff turnover is frequent in this context, using champions as an implementation strategy should be re-evaluated and adapted continuously, which may involve continuous training and replacement of champions or possible incentives to enable champions to stay in these roles.

#### The Role of the Inner and Outer Setting

The inner setting encompasses the structure and culture of the primary care clinics where the teleophthalmology program was implemented, whereas the outer setting connotes the external effects on the implementation process, such as patient needs and resources as well as external policies and incentives [39]. Our findings indicate that the clinical staff thought favorably of the implementation of the teleophthalmology program as it improved the quality of life and quality of care for patients with diabetes, which resulted in a more receptive implementation atmosphere [40]. These results are in parallel with the evidence suggesting that improving organizational receptivity toward change has a direct and positive correlation with the adoption, implementation, and sustainability of programs [41]. However, the lack of organizational incentives and substantial increase in burden and responsibilities for nurses negatively affected readiness and receptivity in our clinics. Unlike physicians, who may be paid based upon the pay-for-performance model, similar financial incentives did not exist for nurses in the primary care clinics. Thus, for nurses, the implementation of programs such as teleophthalmology may lead to an extra workload without concurrent proper increase in remuneration or other incentives or rewards.

Our study found that adjusting the primary care clinic workflow might be needed to successfully integrate the teleophthalmology program. Initially, staff felt that the intervention tasks did not fit well within their existing workflow when first introduced. In addition, the limited physical space of the clinics may have compounded the issue with the workflow. However, when clinic staff were given the ability to develop solutions to better modify the processes of the teleophthalmology intervention to allow it to become more seamless in the clinic workflow, they were more accepting and willing to carry out the process. Liu et al [18] also found that placing an excessive burden on the clinical personnel, as well as high staff turnover and insufficient staffing can directly result in a decrease in clinical personnel satisfaction and may also jeopardize the viability and success of teleophthalmology programs in the primary care setting. They stressed the importance of engaging with clinic staff to make mutually informed changes in the program to ensure that the program properly fits into the existing workflow [18]. Studies have shown that time and space constraints along with disruption of existing, well-ingrained processes are the main obstacles to fit a new program into the workflow [42]. Moreover, there are a variety of views regarding managing the workflow to successfully implement interventions [42]. One view states that interventions should be adjusted accordingly to fit into the predefined workflow. An opposing view suggests that alterations in the workflow are unavoidable and fundamental for the program to successfully achieve its goals [42]. These 2 drastically different opinions highlight the fact that, despite the consequential effect of workflow on the success of an intervention, there still appears to be no definitive approach to workflow standards.

Learning from experience and from the results of this study, our teleophthalmology program was modified to better integrate it with the existing workflow in the primary care setting. The space constraint was addressed by moving the camera to a clinic room only on the days of the week designated for carrying out the teleophthalmology program. This strategy allowed for flexibility in scheduling appointments and accessibility to the camera throughout the day. Furthermore, the electronic intake form was shortened and made easier to fill out on the web platform used for the teleophthalmology program. In addition, staff were asked to capture only 50% (2/4) of the images per eye (macular centered and anterior segment image) to reduce the time spent photographing the patients’ eyes. These changes decreased the time to complete the teleophthalmology-based evaluation and increased its acceptance by staff and patients. This increased the number of patients evaluated through teleophthalmology. Finally, to ensure that the added responsibility of the intervention did not burden the nurses, primary care leadership suggested assigning and training data coordinators instead of nurses to carry out the teleophthalmology workflow. In doing so, the nurses were freer to attend to tasks that needed their skill set, which allowed for more efficient use of clinic resources. Web-based training modules on operating the camera and assessing visual acuity were made available to all staff participating in the teleophthalmology workflow to accommodate such transition and promote staff education. These changes have increased adoption of teleophthalmology by more primary care clinics in the health system and have increased the number of patients evaluated with teleophthalmology in currently participating clinics.

We also interviewed staff on their knowledge of the costs of the program to the patient and the financial feasibility of the program through financial incentives from meeting quality metrics and through fee-for-service insurance billing. Emphasis on constructing a sustainable business model was not the focus of the piloting of the program in the 3 clinics, and most of the staff we interviewed were not involved in insurance billing. Only 33% (1/3) of the clinics performed fee-for-service billing for the program. We were only able to interview 1 program administrator and a few physician leaders who knew more about the financial aspect of the program. Some did acknowledge that insurance reimbursement may not be enough to make the intervention sustainable as reimbursement for ophthalmic photography was inconsistent among the various insurers, especially the government-funded insurers Medicare and Medicaid, which insured most of the safety-net patient population [22]. In our program, Medicare did not reimburse for instances where the photographs did not show any pathology. There are different limitations and guidelines in Medicaid programs for each state, and each regional Medicare governing body has their own rules for coverage of telemedicine interventions [43]. The uncertainty around reimbursement for this service is seen as a potential barrier to wide-scale implementation of teleophthalmology in the primary care setting [43].

In our study, nurses indicated that patients with diabetes had little knowledge of the severe impacts of diabetes on eye health, which reduced their adherence to eye care. In addition to lack of knowledge, several factors may affect patients’ adherence. The absence of necessary information and recommendations about the significance of preventive eye health screening by the primary care providers to their patients is a known barrier to seeking timely preventive eye care [44]. Furthermore, the absence of primary care recommendations leads to low perceived vulnerability to diabetic retinopathy in patients with diabetes [44]. An et al [45] conducted a retrospective study to evaluate the long-term adherence of Americans with diabetes to the recommended retinal screening. They reported that patients with low socioeconomic indicators (income and educational attainment) and low diabetes-related health education were less likely to have annual dilated eye examinations [45]. They also determined an inverse association between the specialist’s copayment and the patient’s adherence to eye examinations [45]. Implementing teleophthalmology in primary care clinics as a convenient and affordable addition to routine primary care visits can potentially address many of the mentioned barriers to eye care, particularly for those at risk of missing their recommended eye care appointments [46]. However, lack of knowledge and negative or even indifferent attitudes toward teleophthalmology by primary care providers can be considerable barriers to its integration and effectiveness. Most patients with diabetes have still not heard about telemedicine. Their willingness to take part in teleophthalmology is often contingent upon their relationship with their primary care provider, their health status, the cost of receiving such care, and their opinion on its convenience [47].

### Strengths and Limitations

This study focused on both provider and nursing staff, who were the ones to actually carry out the workflow of the teleophthalmology program. It identified the factors influencing adoption and use of teleophthalmology in urban primary care safety-net clinics with a large racial and ethnic minority population in 1 city. Consequently, the generalizability of the findings is limited to that population. As only 1 administrator at 1 clinic was interviewed, the perspectives presented in this study may not fully reflect the experience of administrative staff (who were responsible for coding and billing and were probably more familiar with the complexities of financial reimbursement), ophthalmologists, or patients (who were the focus of another published study [28]). Moreover, there are several factors beyond the clinic level that may affect the success of implementation, many of which are related to patients’ needs and experiences (which are not the focus of this study) as well as the broad financial context of health care in the United States, which was not brought up by the participants. Further study of these elements to fully understand the factors leading to successful implementation of teleophthalmology for diagnostic eye care in primary care settings is needed.

### Conclusions

Overall, in our study, primary care staff expressed that having a teleophthalmology program for patients with diabetes in their clinics was valuable. Ensuring standardization of processes, workflows, and knowledge among staff and patients; having adequate staff, space, and time; consistently well-functioning technology with robust customer support; financial viability (including understanding of the impact on patient finances); and continuous engagement with care coordination between primary care and eye care to improve timely follow-up to eye care are needed for ideal implementation.

## Appendix

Multimedia Appendix 1 Qualitative interview guide.

## Abbreviations

CFIR: Consolidated Framework for Implementation Research
DRS: diabetic retinopathy surveillance
NP: nurse practitioner
PRISM: Practical, Robust Implementation and Sustainability Model
RN: registered nurse
