# Supplementary material for: Implementation of Teleophthalmology to Improve Diabetic Retinopathy Surveillance: Qualitative Interview Study of Clinical Staff Informed by Implementation Science Frameworks
Source: JMIR Diabetes. 2022 Mar 30;7(1):e32162. doi: 10.2196/32162 (PMC9008535; doi:10.2196/32162)
Supplement: Multimedia Appendix 1 [file diabetes_v7i1e32162_app1.docx]

Non-italics: Adapted from CFIR

Italics: Adapted from PRISM

What is the program in your words/setting?

- Can you briefly describe the camera screening intervention in your own words?

Why is the intervention being implemented? (what is the need?)

- Why was the intervention started in your clinic?

What information was provided to convince you, key stakeholders, that intervention would work and address your need?

- How was the camera screening service originally explained and what types of information was provided to the staff?
- When learning about the intervention, was there sufficient buy-in from the clinic staff (e.g. providers, nurses, admin staff)? And if not, what would have been useful to get more buy-in?

How does the intervention compare to other similar/existing programs or alternative methods to address the problem (low number of diabetics having retinal exams)?

- Have there been other telehealth programs in the clinic? If so, how does this intervention compare?

*How has the program been embedded in workflow?*

- *If problems arose with the intervention, how was the clinic staff able to address them?*

How does the intervention compare to other similar existing programs in your setting?

- Have there been other telehealth programs in the clinic? If so, how does this intervention compare?

What supports, such as online resources, marketing materials, or a toolkit, are available to help you implement and use the intervention?

- Before starting the intervention what support material such as online resources, marketing materials, or a toolkit would have helped make the implementation roll out smoother?

What costs will be incurred to implement the intervention?

- Currently, how much concern is there about overall cost of the program in regard to long-term sustainability?

*Is the program complex and/or costly for patients?*

- *How much does the screening cost for patients with and without insurance, and do you feel that the cost of the screening influences the willingness of patients to be screened?*

Have you elicited information from participants regarding their experiences with the intervention?

- What do the patients seem to like and dislike about the service, how willing were they to participate? (receptive)
- What feedback did the clinic staff get from patients after they participated in the study?

*Do reimbursement or coverage issues affect patient or staff behavior?*

- *How much does the screening cost for patients with and without insurance, and do you feel that the cost of the screening influences the willingness of patients to be screened?*

What do you know of other organizations implementing the intervention or similar programs?

- Have you heard about similar programs in other clinics?

*Should the sustainability infrastructure be the same as that used for implementation?*

- *What type of resources would the clinic need to move from implementing the intervention to making it sustainable?*

*Is there an existing infrastructure that can take on key implementation/ sustainability tasks?*

- *What infrastructure features are needed to expand this program to other clinics in the Primary Care Network?*
- *If the intervention was brought back to the clinic as a service, could the clinic more effectively sustain the camera screening?*

*Do key staff expect program to be sustainable?*

- *How did the clinic staff view the sustainability of the intervention?*

*How do staff at all levels perceive net benefit of program?*

- *How did the clinic staff perceive the benefit of the intervention? Did the clinic staff feel the intervention was beneficial to the patient and clinic overall?*

What kinds of infrastructure changes will be needed to accommodate the intervention?

- What has the clinic staff been able to address problems with the camera screening service (e.g. negative effects on work flow)?
- What changes to the work flow the clinic would have made the implementation of the intervention easier?

How confident do you think your colleagues feel about using the intervention?

- How confident do you feel the clinic staff felt with the intervention such as using the camera?

What is the general level of receptivity in your organization to implementing the intervention?

- How receptive was the clinic staff to learning to screen patients, such as assessing vision and using the camera, and how confident were they in performing these tasks as a part of this program?

*Will the intervention replace or compliment a current program or process?*

- *Before the intervention was implemented, how did the clinic try to help diabetics get eye care? Now that the intervention has finished, how does the clinic try to help diabetics get eye care?*

*Can you describe how the intervention will be integrated into current processes?*

- *How has the screening been integrated into the standard PCP visit and what workflow changes were necessary?*
- *How was the screening incorporated into the clinic visit?*

*How well does the intervention fit with existing work processes and practices in your setting?*

- *How well did the intervention fit into the clinic set up, were a lot of changes needed?*

*To what extent does your organization/unit set goals for current programs/initiatives?*

- *What are the programs current goals?*

*What are some barriers for getting your eyes screened with the camera?*

- *Why did some patients decide not to get their eyes screened?*

*What are some benefits for getting your eyes screened with the camera?*

- *What do the patients seem to like and dislike about the service, how willing were they to participate? (receptive)*
